# Monocyte-Derived Dendritic Cell Differentiation in Inflammatory Arthritis Is Regulated by the JAK/STAT Axis via NADPH Oxidase Regulation

**DOI:** 10.3389/fimmu.2020.01406

**Published:** 2020-07-07

**Authors:** Viviana Marzaioli, Mary Canavan, Achilleas Floudas, Siobhan C. Wade, Candice Low, Douglas J. Veale, Ursula Fearon

**Affiliations:** ^1^Molecular Rheumatology, School of Medicine, Trinity Biomedical Sciences Institute, Trinity College Dublin, Dublin, Ireland; ^2^Rheumatology EULAR Centre of Excellence, Centre for Arthritis & Rheumatic Diseases, St Vincent's University Hospital, University College Dublin, Dublin, Ireland

**Keywords:** monocyte-derived dendritic cells, differentiation, inflammatory arthritis, tofacitinib, NADPH oxidase

## Abstract

Monocyte-derived Dendritic cells (Mo-DC) are a distinct DC subset, involved in inflammation and infection, they originate from monocytes upon stimulation in the circulation and their activation and function may vary in autoimmune diseases. In this study we investigate the differences in Mo-DC differentiation and function in patients with Rheumatoid (RA) compared to Psoriatic arthritis (PsA). A significant increase in the Mo-DC differentiation marker CD209, paralleled by a corresponding decrease in the monocytic marker CD14, was demonstrated in RA compared to PsA, as early as 1 day post Mo-DC differentiation. RA monocytes *ex-vivo* were phenotypically different to PsA, displaying a more mature phenotype associated with altered cellular-morphology, early dendrite formation, and a significant increase in the CD40 marker. In addition, SPICE algorithm flow cytometric analysis showed distinct differences in chemokine receptors distribution in HC compared to PsA and RA CD14^+^ cells in the blood, with increased expression of the chemokine receptors CCR7 and CXCR4 observed in PsA and RA. In addition CD14^+^ cells at the site of inflammation showed a different chemokine receptor pattern between PsA and RA patients, with higher expression of CXCR3 and CXCR5 in RA when compared to PsA. The early priming observed in RA resulted in monocyte-endocytosis and antigen-uptake mechanisms to be impaired, effects that were not observed in PsA where phagocytosis capacity remained highly functional. Tofacitinib inhibited early Mo-DC differentiation, decreasing both CD209 and CD40 activation markers in RA. Inhibition of Mo-DC differentiation in response to Tofacitinib was mediated *via* an imbalance in the activation of NADPH-oxidases NOX5 and NOX2. This effect was reversed by NOX5 inhibition, but not NOX2, resulting in suppression of NOX5-dependent ROS production. In conclusion, RA monocytes are already primed *ex vivo* to become DC, evident by increased expression of activation markers, morphological appearance and impaired endocytosis capacity. Furthermore, we demonstrated for the first time that NOX5 mediates Mo-DC differentiation and function in response to Tofacitinib, which may alter DC functions.

## Introduction

Dendritic cells (DCs) are a heterogeneous population of professional antigen-presenting cells at the interface of innate and adaptive immunity (1, 2). DCs are classified according to their tissue location and function (3), a specific subset of DCs derived from monocytes (Mo-DC) have a key role in inflammation (4) and infection (5), often termed “inflammatory Mo-DC” (6). Although the distinct role and function of Mo-DC is still under investigation, due to the rarity and lack of definitive markers, it is widely accepted that inflammatory Mo-DC play a complementary role to conventional DC (CD141 and CD1c) to synergize with them in response to inflammation or infection (3, 6). While conventional DCs are critical for self-tolerance and for triggering specific immunity, inflammatory DCs are mainly involved in innate defenses and T-cell activation (7). Monocyte differentiation into DC is driven by multiple signals, including NADPH oxidases (NOX) which play a key role in both Mo-DC differentiation and antigen presentation, with NOX5 identified as a key regulator of Mo-DC development in healthy subjects (8).

Inflammatory arthritis, including Rheumatoid arthritis (RA) and Psoriatic arthritis (PsA) are chronic autoimmune diseases characterized by neo-angiogenesis, immune cell infiltration to the synovium and fibroblast-like synoviocyte expansion, leading to bone and cartilage destruction. While the two diseases have many common clinical manifestations, there are significant pathogenic differences at a clinical, anatomical, cellular and molecular level, that may explain differential disease outcomes and prognoses (9). An increasing number of studies has sought to address the role of DCs in the pathogenesis and progression of RA and PsA. Most studies have shown a decrease in circulatory DC which display an immature phenotype (10–16). In RA, circulatory levels of DC inversely correlate with disease activity, an effect reversed in RA responders (16). Abundant expression of DC have been observed in the synovium of RA and PsA patients in comparison to osteoarthritis (11, 17), with studies demonstrating their ability to guide differentiation of naive T cells in an antigen-specific manner (18). In animal models, presentation of collagen-derived peptides by mature DCs is sufficient for the induction of RA in mice (19), underlying the key role of DCs in the initiation of RA pathogenesis. Mo-DCs, identified as MHCII^+^CD11c^+^, are significantly reduced following anti-GM-CSFRα mAb treatment in animal models of arthritis (20). In turn, CD4 T-cells producing GM-CSF can stimulate monocyte differentiation into an inflammatory subset of DC (CD1c^+^CD16+) in RA synovial fluids (21), suggesting an important role within the joint. However, to date few studies have examined Mo-DC in inflammatory arthritis in relation to disease pathogenesis, disease progression or therapeutic response.

The intricate network of cytokines, involved in the pathogenesis and progression of RA and PsA, activate different molecular pathways, one of which is the Janus Kinase (JAK) family of receptor associated tyrosine kinase (22–24). Activation of JAKs induces signal transducer and activator of transcriptions (STATs), leading to the activation of multiple genes that are involved in driving joint inflammation. Tofacitinib, an oral JAK1 and JAK3 inhibitor (with functional specificity over JAK 2), has recently been approved for the treatment of both RA and PsA (25, 26), however few studies have examined its mechanistic effect in cells or tissue from patients with inflammatory arthritis. Studies to date have demonstrated Tofacitinib inhibits pro-inflammatory cytokine secretion, cellular invasion and metabolic pathways in RA/PsA explants and fibroblasts (27–29). In addition, Tofacitinib has been shown to reduce the T cell stimulatory capacity of human Mo-DC in healthy subjects (30), however the effect of JAK/STAT inhibition on Mo-DC differentiation and function in inflammatory arthritis, such as RA and PsA remains to be elucidated.

In this study we demonstrate that RA monocytes are already primed in the blood to differentiate into DC, evident by the observed faster differentiation rate, early activation and impaired phagocytosis observed in RA compared to PsA. In addition, we demonstrate that the Tofacitinib therapeutic approach targets Mo-DC differentiation by altering the NADPH oxidase balance in DC in favor of NOX5.

## Methods

### Patient Recruitment and Sample Collection

Blood from patients with active inflammatory arthritis (RA *n* = 22, PsA *n* = 17) were recruited from the Rheumatology Department, St. Vincent's University Hospital. Healthy blood, used as a comparison, was obtained from anonymous healthy donors (HC) or Buffies from St. Vincent's University Hospital or St James Hospital. Blood samples from both HC and patients were collected in lithium heparin tubes. Synovial fluid was obtained at arthroscopy or rheumatology clinics (St. Vincent's University Hospital and Tallaght Hospital, Dublin, Ireland). All subjects gave fully informed written consent as approved by the institutional ethics committee and all research was performed in accordance with the Declaration of Helsinki. Patient's demographics are summarized in Supplementary Table 1.

### Cell Isolation and Mo-DC Differentiation

Peripheral blood and synovial fluid -mononuclear cells (PBMC and SFMC, respectively) were isolated by density gradient centrifugation (Lymphoprep, Stemcell Technologies) according to the manufacturer's recommendations. Peripheral blood mononuclear cells were washed and suspended in MACS buffer and CD14^+^ monocyte purified by magnetic positive isolation with the CD14 MicroBeads (Miltenyi Biotec) following manufacturer's instructions. Monocytes were plated in RPMI 1640 media supplemented with 10% Hyclone fetal bovine (Thermo Fisher Scientific), streptomycin (100 units/mL) (Sigma-Aldrich) and penicillin (100 units/mL) (Sigma-Aldrich) and stimulated with 70 ng/mL granulocyte-macrophage colony stimulating factor (GM-CSF) (PeproTech) and 50 ng/mL interleukin-4 (IL-4) (PeproTech) for 7 days in order to obtain immature Mo-DC as described previously (31). For time-course differentiation experiments, cytokines were added for 1, 2, or 3 days. To evaluate the effect of Tofacitinib (Pfizer) on Mo-DC differentiation, cells were treated with 1 μM Tofacitinib or DMSO vehicle control for 15 min prior to the cytokine stimuli. For the NOX inhibitor studies, the NOX5 inhibitor Celastrol (1 or 2 μM CEL-R&D System) and the NOX2 inhibitor gp91 ds-tat Peptide 2 (15 μM TAT- Cambridge bioscience) were added 5 min before Tofacitinib treatment (0.5 or 1 μM), followed by cytokines stimuli. The Tofacitinib concentrations utilized are based on ours and others previous studies, which showed no effect on cells viability (27, 30).

Cell length was measured with Image J. A minimum of 10 cells per field were randomly selected from three different images and the length was measured by analyzing the length of the straight segment line along the length of the single cell and then quantified by ROI manager Image J program. The average of the readings is represented as Mean ±SEM.”

### Flow Cytometry

Differentiation of Mo-DC were analyzed by flow cytometry. Cells were gated based on forward and side scatter and dead cells and doublets were removed. Live Dead Red or Near IR (Molecular Probes) was used to eliminate dead cells. To eliminate non-specific binding of mouse monoclonal antibodies to Fc-gamma receptor (FcγR), samples were blocked with a human FcγR-binding inhibitor prior to antibody staining (BD Bioscience or Biolegend). The following antibodies were used in combination to assess Mo-DC differentiation and maturation and to identify the CD14^+^ population: CD209 FITC (BD Bioscience) or PerCP/Cy5.5 (Biolegend) (Clone DCN46), CD14 PE (Biolegend) or Brilliant Violet 510 (Biolegend) (Clone M5E2), CD86 FITC (BD Bioscience) (Clone 2331), CD80 PE (BD Bioscience) (Clone L307.4), CD40 PE/CY7 (Biolegend) (Clone 5C3), HLA-DR Brilliant Violet 785 or 421 (Biolegend) (Clone G46-6) and CD11c APC/CY7 or PerCP/Cy5.5 (Biolegend) (clone Bu15), CD45 FITC (BD Bioscience) (Clone HI30), Lineage (LIN) APC (Biolegend) (CD3/CD56/CD19/CD20, clones UCHT1; HIB19; 2H7; 5.1H11), CCR6 Brilliant Violet 711 (Biolegend) (Clone G034E3), CCR7 PE/DAZZLE (Biolegend) (Clone G043H7), CXCR3 Brilliant Violet 650 (Biolegend) (Clone G025H7), CXCR4 PE/CY5 (Biolegend) (Clone 12G5), CXCR5 Brilliant Violet 786 (Biolegend) (Clone J252D4) antibodies. Mo-DC Cells were gated for CD11c. The CD14^+^ cell population in PBMC and SFMC were gated as CD45^+^/LIN^−^(CD19-CD20-CD56-CD3)/HLA-DR^+^/CD14^+^, as previously reported (32, 33). Chemokines receptor expression was identified by comparison with an FMO (Fluorescence Minus One Control). Samples were acquired using the CyAn or Fortessa Flow Cytometer (Beckman Coulter) and analyzed using Flowjo software (Treestar Inc.).

### Antigen Uptake

Antigen uptake was evaluated by flow cytometry using a well-established methodology previously described (34, 35). Briefly, Mo-DC were harvested and washed in phosphate-buffered saline (PBS). The resulting pellet was re-suspended in completed medium and transferred to flow-cytometry tubes containing either 4 μl DQ^TM^ Ovalbumin (DQ OVA, Molecular Probes) or 6 μL Lucifer Yellow dilithium salt (LY, Sigma-Aldrich). Tubes were incubated in parallel at 4 and 37°C for 15 min (DQ OVA) or 120 min (LY) and washed twice in cold FACS buffer. The incorporated fluorescence of both the fluorescent reporters DQ-OVA (receptor-mediated endocytosis) and LY (fluid-phase endocytosis) was analyzed by flow cytometry, cells were excited with the 488 nm laser and fluorescence was detected using the 530/30 bandpass filter. A comparison between the histograms and corresponding mean fluorescence intensities (MFI) between cells incubated at 37°C (specific uptake) and cells incubated at 4°C (non-specific uptake). Fluorescence-based techniques have been previously used to monitor endocytosis in monocytes and dendritic cells, in the presence of NOX (36–38), although it is important to consider the possible quenching effect of excessive ROS production on the fluorochrome.

### Protein Expression

Protein expression was evaluated by western blot, using standard sodium dodecyl sulfate–polyacrylamide gel electrophoresis (SDS-PAGE) techniques as previously described (8). Briefly, harvested cells were lysed in 5X Laemmli sample buffer (5 mmol/L Na3VO4, 2.5 mmol/L p NPP, 10 mmol/L NaF, 5 mmol/L EDTA, 5 mmol/L EGTA, 20 μg/L leupeptin, 20 μg/L pepstatin and 20 μg/L aprotinin) and incubated for 3 min at 70°C and stored at −80°C until use. Lysates were sonicated and subjected to 10% SDS-PAGE. The separated proteins were transferred to nitrocellulose and blocked in 5% milk in Tris-buffered saline containing Tween 20 (TBS-T) for 1 h. The membranes were then probed overnight with primary antibodies-anti-gp91/NOX2 (Santa Cruz 1:5,000), anti-NOX5 (Abcam 1:1,000), and anti-actin (Sigma 1:10,000). Membranes were washed and incubated with HRP-labeled specific antibody. The signal was detected using SuperSignal® West Pico Chemiluminescent Substrate (Amersham Biosciences) and density of the bands was analyzed using EDAS 120 system from Kodak (Kodak, Rochester, NY, USA).

### Reactive Oxygen Species (ROS) Production

To determine cellular ROS release from Mo-DC in the presence of Tofacitinib, DCFDA Cellular ROS Detection Assay Kit (Abcam, UK) was utilized. Monocyte were seeded and differentiated for 48 h as described previously (8). Cells were detached, washed and stained with 10 μM DCFDA in 1 × buffer for 30 min at 37°C and 5% CO_2_. Cells were washed and seeded into clear bottom, dark sided 96-well plates at a density of 1 × 10^5^ cells/well. Cells were treated with Tofacitinib or DMSO control for 20 min and then treated with 1 μM Ionomycin to induce NOX5- dependent ROS. ROS was measured using the Spectra Max Gemini System with excitation and emission wavelengths of 485 and 535 nm, respectively. Mean fluorescence values in duplicate for each condition were obtained.

### Statistical Analysis

Data were analyzed with the GraphPad Prism 7 software. Differences between groups were analyzed by the 1-way analysis of variance (ANOVA) test with Tukey multiple comparison post-test, two-way ANOVA with Bonferroni and Tukey multiple comparison or Multiple *t*-test, corrected for multiple comparisons using the Holm-Šídák method. ^*^p <0.05, ^**^p < 0.01, and ^***^p < 0.001 values were considered as significant.

## Results

### Monocyte-Derived Dendritic Cells From RA and PsA Patients Differentiate at a Similar Rate to HC

**Figure 1:**
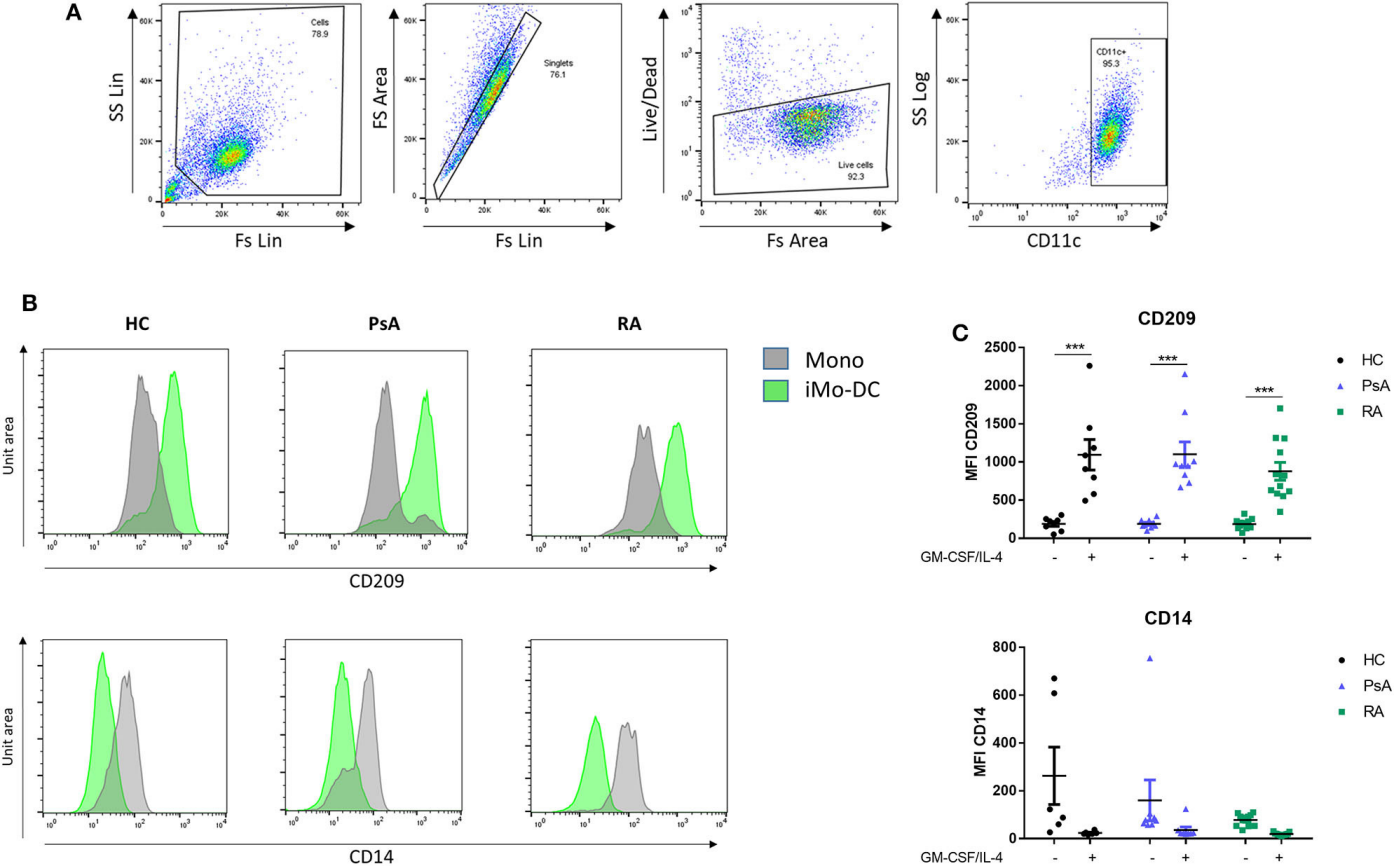
Mo-DC differentiation in RA and PsA patients. Monocytes from HC, PsA, or RA patients were differentiated for 7 days with GM-CSF/IL-4. **(A)** Gating strategy: Cells were gated, doublets and dead cells excluded. Cells were gated for CD11c expression. **(B)** Representative histograms and **(C)** dot-plot representation of at least 3 separate experiments (n HC = 8, PsA = 9, RA = 12) of CD209 (Top), Mo-DC surface differentiation marker and CD14 (bottom), monocyte markers were evaluated by flow cytometry in the CD11c^+^ population. Data are represented as mean ±SEM and differences among groups were evaluated by two-way ANOVA with Bonferroni post-test. *p < 0.05, **p < 0.01, ***p < 0.001. SS, side scatter; FS, forward scatter; Lin, linear scale.

RA, PsA, and HC monocytes were differentiated for 7 days into monocyte-derived dendritic cells (Mo-DC), and analyzed by flow cytometry. Mo-DC were gated on CD11c^+^ following exclusion of doublets and dead cells, as shown in Figure 1A (representative gating strategy). CD209 and CD14 surface expression were evaluated. Flow cytometric analysis of HC, PsA and RA monocytes (representative histograms Figure 1B), show similar marked and statistically significant increases in the expression of the differentiation marker CD209 for all groups following 7 days stimulation with GM-CSF/IL-4 (*p* < 0.001) (Figure 1B), paralleled by a reduction in CD14 expression (Figure 1C).

### RA Monocyte Are Primed to Differentiate Into Dendritic Cells

**Figure 2:**
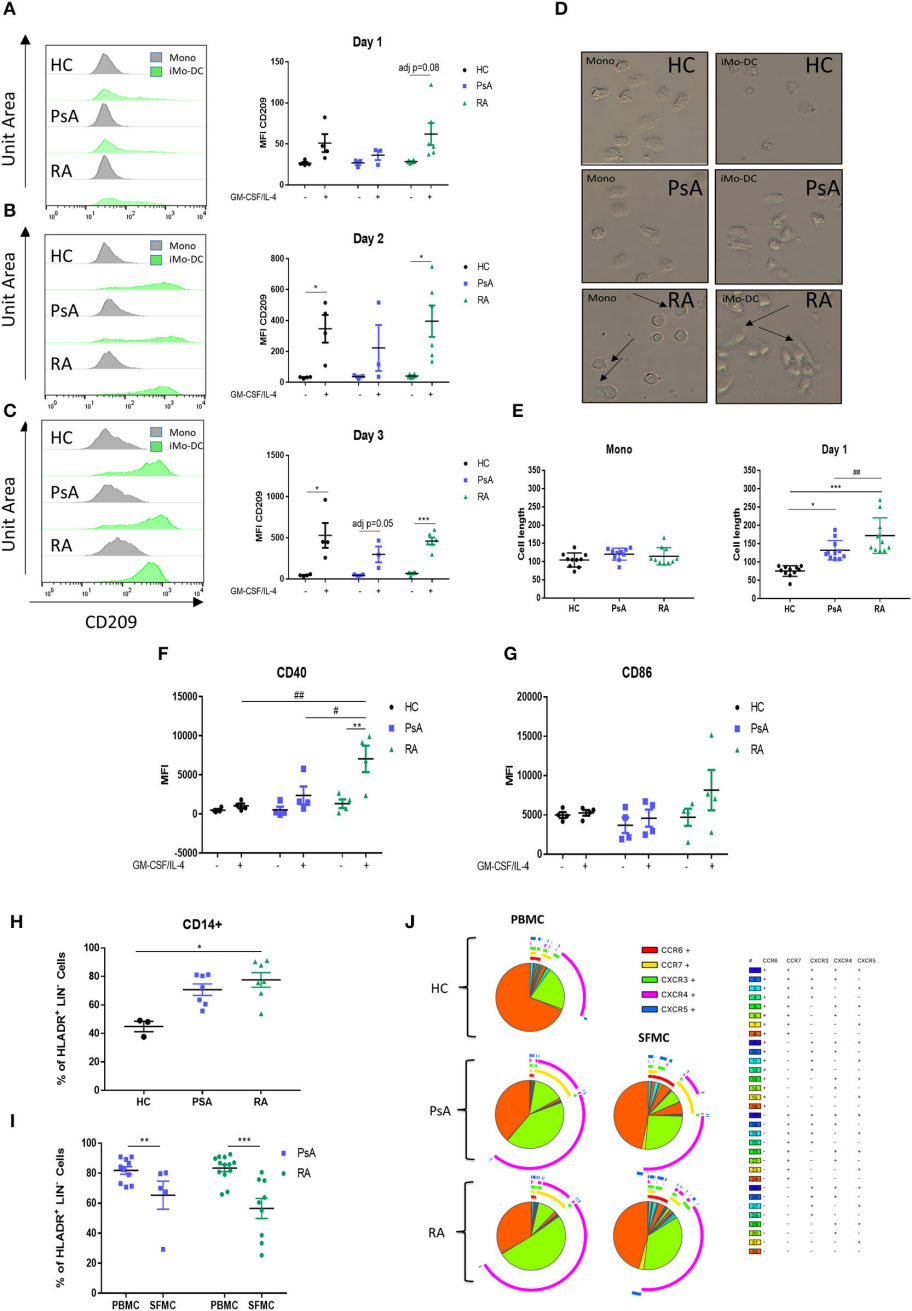
RA monocytes are primed to differentiate into DC. Monocytes from HC, PsA or RA patients were differentiated 1–2 and 3 days with GM-CSF/IL-4. CD209 surface expression was analyzed by flow cytometry in the CD11c+ population at **(A)** 1 day, **(B)** 2 days, and **(C)** 3 days post GM-CSF/IL-4 stimulation. Left, representative histograms; Right, average of at least three separate experiments (*n* HC = 4, PsA = 3, RA = 6). Differences among groups were calculated with non-parametric Multiple t-test, corrected for multiple comparisons using the Holm-šídák method. The adjusted *p*-values are here represented (*p < 0.05, **p < 0.01, ***p < 0.001). **(D,E)** Monocytes from HC, PsA or RA patients were differentiated for 1 day with GM-CSF/IL-4. **(D)** Representative microscopic images (Leica inverted microscope 40X, arrows indicate elongated cells). **(E)** Cell length quantification. Ten to twelve cells were measured from three different images. **(F,G)** Maturation surface markers **(F)** CD40 and **(G)** CD86 were investigated by flow cytometry in the CD11c+ population (HC/PsA/RA = 4). **(H)** CD14+ population in PBMC from HC (*n* = 3), PsA (*n* = 7), and RA (*n* = 7). Gating strategy in Supplementary Figure 1I, CD14+ population in PBMC and SFMC from PSA (PBMC *n* = 10, SFMC *n* = 5) and RA (PBMC *n* = 13, SFMC *n* = 9) patients. **(J)** SPICE algorithm flow cytometric analysis of peripheral blood of HC (*n* = 5), PsA (*n* = 9), and RA (*n* = 14) and synovial fluid from PsA (*n* = 5) and RA patient (*n* = 9) CD14+ cell expression of the chemokines receptors CCR6, CCR7, CXCR3, CXCR4, and CXCR5 (dot plot can be found in Supplementary Figure 3). Data are represented as mean ± SEM and differences among groups were evaluated with two-way ANOVA with Bonferroni post-test. *p < 0.05, **p < 0.01, ***p < 0.001 and (differences between Mo-DC with and without TOFA within the same group), ^#^*p* < 0.05, ^*##*^*p* < 0.01, ^*###*^*p* < 0.001 (differences between HC, RA, and PsA samples).

We next sought to evaluate how early Mo-DC differentiation occurred in each group, thus CD14^+^ monocytes from RA, PsA, and HC were stimulated as described above and acquired by flow cytometry at 1, 2, and 3 days following GM-CSF/IL-4 stimulation. Representative histogram are shown in Figures 2A–C. Interestingly, we observed that the differentiation rate of RA monocytes was faster than that of PsA and HC groups. One day post-stimulation we observed a trending increase in CD209 expression in RA monocytes (adj *p* = 0.08) (Figure 2A). By day 2 both HC and RA showed a significant increase (Figure 2B, adj *p* < 0.05), with no effect observed in monocytes from PsA patients. At day 3 CD209 expression in RA monocytes was further increased (Figure 2C adj *p* < 0.001), while PsA monocytes showed a trending increase (adj *p* = 0.05). The limited number of PsA patients may explain the lack of significance at day 3. The observation that GM-CSF receptor was demonstrated to be increased in CD14^+^ cells in RA (*p* < 0.05), and to a lesser extent, in PsA patients (Supplementary Figures 2A,B), suggested that the priming of RA monocytes was not due to an increase in the cytokine receptor.

In parallel, morphologic analysis of RA monocytes *ex vivo* demonstrated early formation of dendrite protrusions even without the differentiation stimuli (Figure 2D; top panel-arrows), whereas PsA and HC monocytes displayed a round inactive morphology. Following 1-day of differentiation this effect was even more pronounced for RA monocytes, where they displayed an elongated phenotype with dendritic spikes, a morphology not observed in PsA or HC monocytes (Figure 2D; middle panels). Quantification of the cell length demonstrated a significant increase in the length of RA monocytes/Mo-DC compared to HC and PsA (*p* < 0.05 and *p* < 0.001 respectively) (Figure 2E), with a significant difference observed between RA and PsA Mo-DC (*p* < 0.01). By day 7 the cell morphology of Mo-D was very heterogeneous and no significant differences were observed within the groups (data not shown). The elongated phenotype at day 1, paralleled by the increase in CD209 differentiation marker observed in RA monocytes, translated into a significant increase in the maturation marker CD40 compared to unstimulated control (*p* < 0.01 Figure 2F), in addition to an increase in CD86 (Figure 2G). Furthermore, CD40 expression was significantly increased in RA monocytes compared to that of PsA and HC (*p* < 0.05 and *p* < 0.01, respectively).

We next evaluated the CD14^+^ population in PBMC from PsA and RA. The cells were gated as CD45^+^/LIN^−^(CD19-CD20-CD56-CD3)/HLA-DR^+^/CD14^+^, as shown in (Supplementary Figure 1). We observed an increase in the CD14^+^ population in RA (*p* < 0.05), and to a lesser extend in PsA, when compared to HC (Figure 2H), suggesting a higher percentage of circulatory CD14^+^ monocytes in the blood of RA and PsA patients. In addition, we investigated the infiltration of CD14^+^ monocytes within the joints of IA patients, and interestingly observed a decrease in the CD14^+^ population in the SFMC of RA patients (*p* < 0.001) and PsA patients (*p* < 0.01) (Figure 2I). This might suggest differentiation of these cells at the site of inflammation, into monocytic-derived cells. In order to evaluate the chemokine receptor expression of these cells, we performed a SPICE algorithm flow cytometric analysis (39) of peripheral blood and synovial fluid RA and PsA patient CD14^+^ cell expression of the chemokine receptors CCR6, CCR7, CXCR3, CXCR4, and CXCR5 (Figure 2J and Supplementary Figure 3). We observed distinct differences in chemokine receptor distribution between HC and IA CD14^+^ cells, with increased expression of the chemokine receptors CCR7 and CXCR4 (Figure 2J, yellow and green arc), both involved in monocyte trafficking and migration (40–42). In addition, when comparing the chemokine receptor pattern of CD14^+^ cells between PBMC and SFMC of both RA and PsA patients, we observed a higher percentage of cells singly expressing and co-expressing chemokine receptors in SFMC compared to that of PBMC. We observed an increase of CXCR3 (*p* < 0.01) and CXCR5 (*p* = 0.05) (Figure 2J green and blue arcs and Supplementary Figure 3B) in the SFMC from RA patients, both of these key chemokine receptors are involved in monocyte and monocyte-derived cell recruitment and retention at the site of inflammation in RA (43, 44). In addition, we observed an increase in the co-expression of CXCR3 and CXCR5 in RA SFMC when compared to the PsA cells.

### Mo-DC From RA Patients Have an Impaired Endocytosis

**Figure 3:**
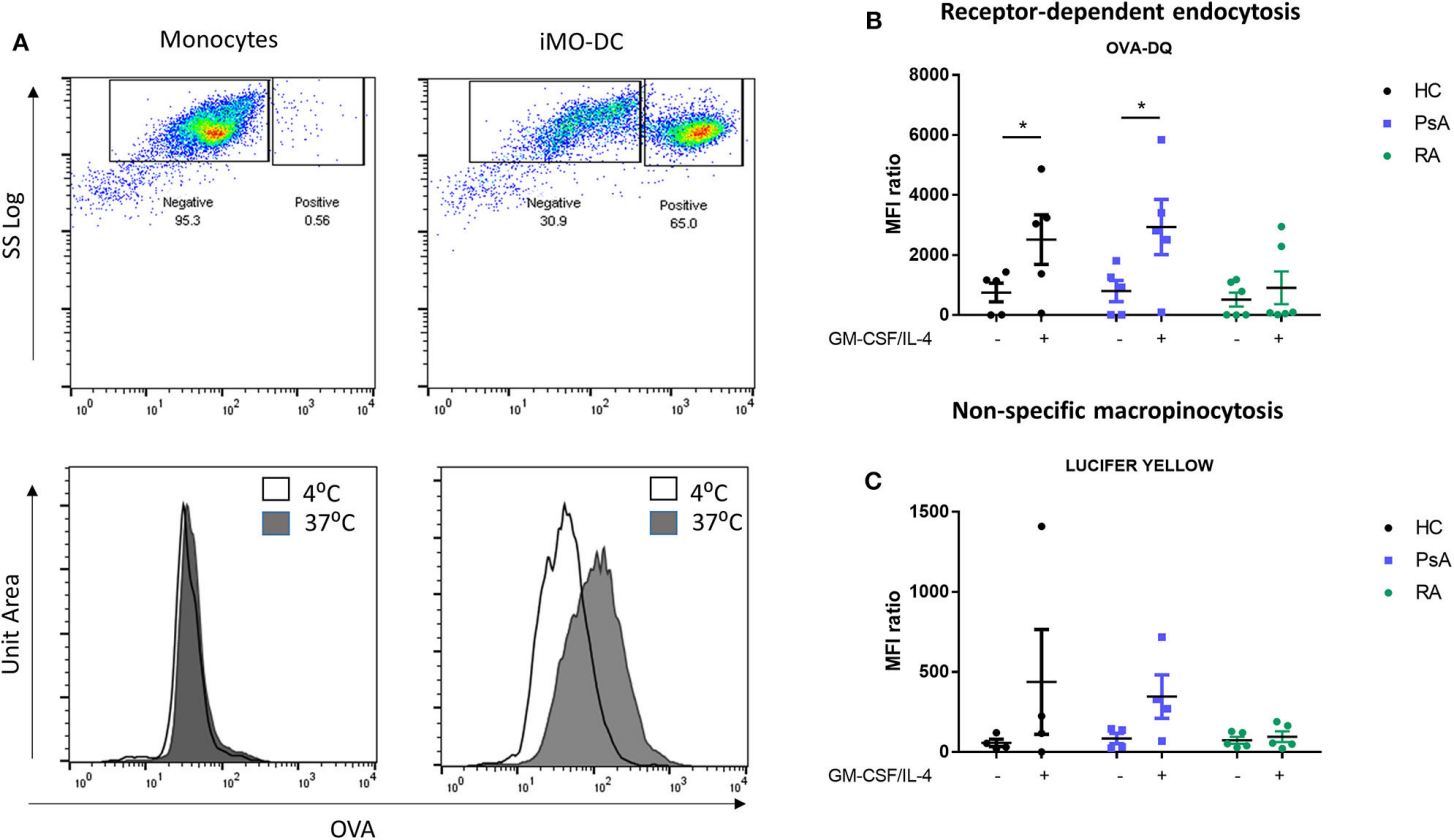
Mo-DC from RA exhibit an impaired endocytosis. Monocytes from HC, PsA, or RA patients were differentiated for 7 days with GM-CSF/IL-4. Endocytosis was evaluated in parallel at 4 and 37°C following antigen incubation. **(A)** Top: Representative plot of Receptor-dependent endocytosis (with OVA-DQ) of cells incubated at 37°C, where the positive population (active endocytosis) can be observed. Bottom: Representative histograms of cells incubated at 4 (white) and 37°C (gray). A clear shift can be observed in immature Mo-DC. Average of at least 3 separate experiments for **(B)** Receptor-depend endocytosis (with OVA-DQ) and **(C)** Non-specific macropinocytosis (Lucifer Yellow) HC (*n* = 4), PsA (*n* = 4), RA (*n* = 6). Data are represented as mean ±SEM and differences were evaluated with two-way ANOVA with Tukey post-test. *p < 0.05.

The main function of Mo-DC in the immature stage is the uptake and processing of antigens (45), therefore we next sought to investigate the possible differences in endocytosis between HC, PsA, and RA Mo-DC. The main endocytic routes in DC are the receptor-mediated endocytosis and the macropinocytosis (31, 46), which were measured by flow cytometric analysis. Representative dot plot and histograms for the receptor-mediated endocytosis are shown in Figure 3A, where a clear increase in OVA (receptor-mediated endocytosis) and a shift in MFI is observed in immature Mo-DC between passive endocytosis (4°C) and active endocytosis (37°C). The quantification of the MRI ratio between active and passive endocytosis demonstrated differences in the response of RA Mo-DC compared to that of PsA and HC Mo-DC. Figure 3B shows a significant increase in the receptor-mediated endocytic activity of PsA and HC Mo-DC (*p* < 0.05), with an increase also observed for non-specific macropinocytosis (Figure 3C). This is in stark contrast to the demonstrated lack of both endocytosis mechanisms observed for RA Mo-DC, suggesting an impaired endocytic activity in these patients.

### JAK/STAT Inhibition by Tofacitinib Alter Mo-DC Differentiation and Function

**Figure 4:**
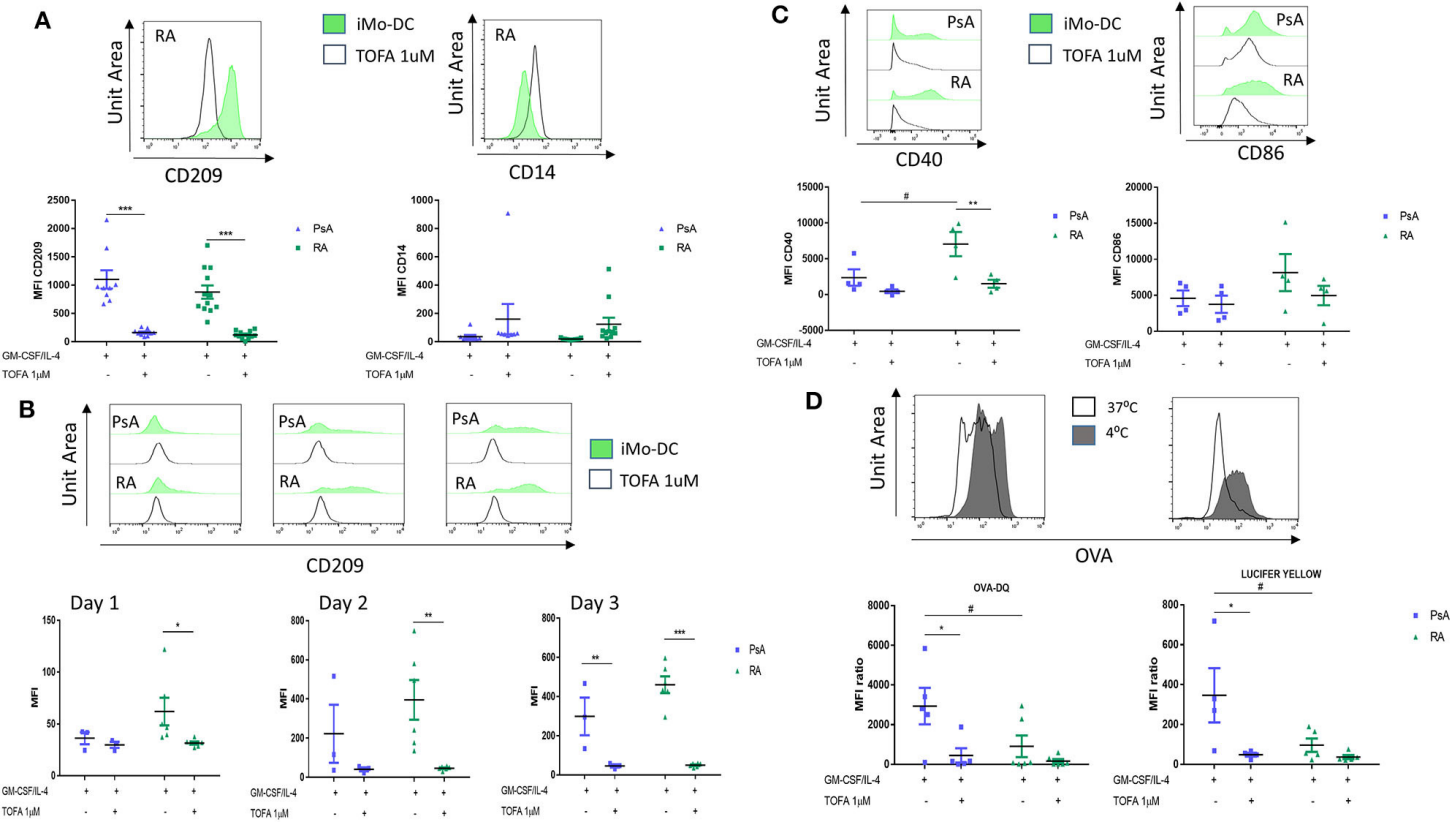
Tofacitinib treatment alter Mo-DC differentiation and function in RA and PsA patients. Monocytes from PsA (*n* = 9) or RA (*n* = 12) patients were differentiated GM-CSF/IL-4 in the presence of absence of Tofacitinib 1 μM (or DMSO). **(A)** 7 day post-differentiation. CD209, surface differentiation marker, and CD14, monocyte markers, were evaluated by flow cytometry in the CD11c^+^ population. **(B)** 1, 2, and 3 days post-differentiation. CD209 surface differentiation marker was evaluated in the CD11c^+^ population in PsA (*n* = 3) and RA (*n* = 6) patients. **(C)** 1 day post-differentiation, CD40 and CD86 activation markers were evaluated in the CD11c^+^ population in PsA (*n* = 4) and RA (*n* = 4) patients. **(D)** 7 day post-differentiation, endocytosis was analyzed as per Figure 3 in Mo-DC from PsA (*n* = 5) and RA (*n* = 6) patients. Data are represented as mean ±SEM and differences among groups were evaluated with two-way ANOVA with Tukey post-test. *p < 0.05, **p < 0.01, ***p < 0.001 (differences between Mo-DC with and without TOFA within the same group) and #*p* < 0.05, ##*p* < 0.01, ###*p* < 0.001 (differences between RA and PsA samples).

It has been previously shown that the JAK/STAT signaling pathway is involved in Mo-DC development (8, 47), and its inhibition is an approved therapeutic strategy for the treatment of both RA and PsA (25, 26). Here we examined the effect of Tofacitinib (JAK1/3 selective inhibitor) on Mo-DC differentiation and function. Monocytes were pre-treated with 1 μM Tofacitinib, 15 min prior to the differentiation stimuli (GM-CSF and IL-4). At the end of the differentiation (day 7), we can observe that JAK/STAT inhibition strongly decreased CD209 surface markers on Mo-DC from both RA, PsA patients and HC (Figure 4A, *p* < 0.001 and Supplementary Figure 4A). This was associated with a lack of decrease of the CD14 monocytic marker, suggesting Tofacitinib inhibits the differentiation of monocytes into DC. This effect occurs very early in the Mo-DC differentiation, as observed by the decrease of CD209 marker in RA patients as early as 1 day after the differentiation stimuli (Figure 4B *p* < 0.05). This effect is then observed by day 3 in both RA and PsA patients (*p* < 0.001 and *p* < 0.01, respectively). This was translated also into a significant inhibition in the activation marker CD40, (Figure 4C, *p* < 0.01), and to a lesser extent CD86, in monocytes from RA patients. Furthermore, Mo-DC from PsA patients lost their endocytic activity, as per receptor-mediated and macropinocytosis, once pre-treated with Tofacitinib (Figure 4D, *p* < 0.05), an effect that was not observed for RA, due the impaired phagocytosis capacity observed (Figure 4D).

### Tofacitinib Alters the NOX2/NOX5 balance in Mo-DC From RA and PsA Patients

**Figure 5:**
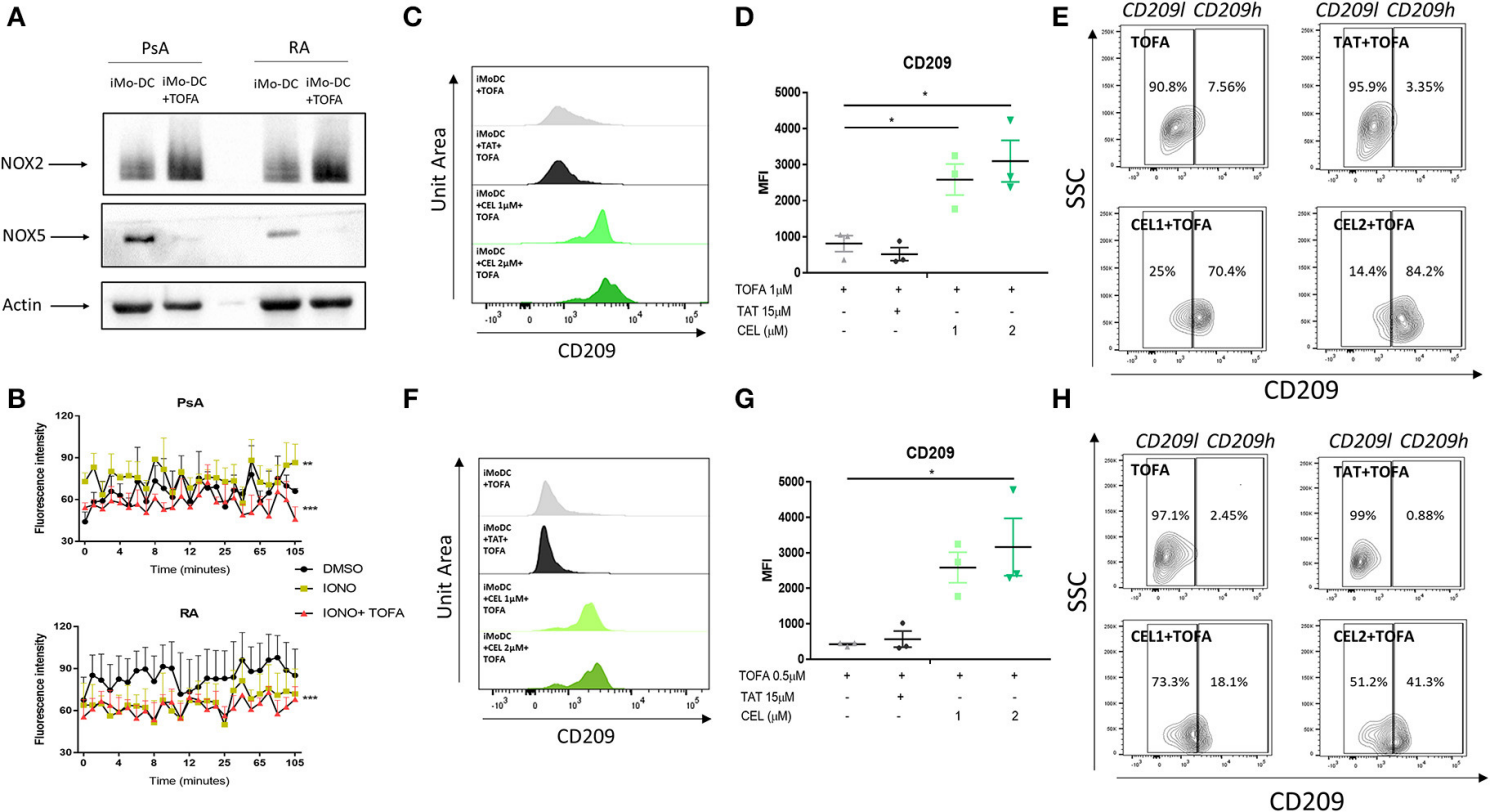
Tofacitinib alters the NOX2/5 balance in PsA and RA patients. Monocytes from HC, PsA or RA patients were differentiated for 7 days with GM-CSF/IL-4 in presence or absence of Tofacitinib 1 μM (or DMSO). Proteins were isolated and western blot for NOX2, NOX5, and B-actin was performed. **(A)** Representative western blots for NOX2, NOX5, and B-actin. **(B)** ROS production in monocytes from PsA (*n* = 3) and RA (*n* = 3) patients differentiated for 48 h and stimulated with 1 μM Ionomycin in the presence or absence of Tofacitinib 1 μM (or DMSO) (Kruskal-Wallis test **p < 0.01 ***p < 0.001 DMSO vs. IONO, ###*p* < 0.001 IONO vs. IONO+TOFA- HC is shown in Supplementary Figure 4B). **(C–E)** HC Mo-DC were stimulated with CEL (1 and 2 μM-NOX5 inhibitor) and TAT (15 μM-NOX2 inhibitor) 5 min before Tofacitinib treatment 1 μM **(C–E)** and 0.5 μM **(F–H)**. **(C,F)** Representative histograms. **(D,G)** Average of HC *n* = 3 (Mean ± SEM) separate experiments. **(E,H)** Representative Contour Plots gating CD209 low (CD209^l^) and CD209 high (CD209^h^) populations. Data are represented as Mean ±SEM. One-Way ANOVA *p < 0.05, **p < 0.01, ***p < 0.001 (differences between Mo-DC with and without TOFA within the same group).

To further investigate the possible mechanism of action by which Tofacitinib inhibits differentiation, we investigated NAPDH oxidase (NOX) protein expression. NOX5 has previously been shown to be increased during Mo-DC differentiation (8). In addition, studies have shown a corresponding decrease in NOX2 expression during differentiation (8). In this study, we demonstrate for the first time the presence of NOX5 and NOX2 in Mo-DC from RA and PsA (Figure 5A). Interestingly, Tofacitinib has the ability to switch this balance, by decreasing NOX5 protein expression and increasing NOX2 protein expression in Mo-DC (Figure 5A). This imbalance was associated with impaired NOX5-dependent ROS production in PsA Mo-DC treated with Tofacitinib (Figure 5B, p < 0.001), which was observed also in HC (Supplementary Figure 4B), whereas a lack of response to Ionomycin was observed in RA cells, leading to a limited effect of Tofacitinib (Figure 5B). Further experiments should confirm these finding in a larger sample size.

The direct effect of NOXs on altering the mechanism of action of Tofacitinib was evaluated by the selective pharmacological inhibition of NOX2 (8, 48) and NOX5 (8, 49). At a concentration of 1 μM Tofacitinib, the presence of the NOX5 inhibitor CEL significantly reduced the effect of Tofacitinib on Mo-DC differentiation, as shown by representative histogram analysis (Figure 5C) and the MFI analysis (Figure 5D, *p* < 0.05 for CEL 1 and 2 μM and Supplementary Figure 4C). A similar effect was observed with lower concentration of Tofacitinib (Figures 5F,G, 0.5 μM *p* < 0.05 for CEL 2 μM and Supplementary Figure 4D). No differences were observed with the NOX2 inhibitor TAT (Figure 5). When analyzing the CD209 high population (CD209^h^) and the CD209 low (CD209^l^) population (Figures 5E,H), we observed that Mo-DC treated with Tofacitinib alone were mostly expressing CD209^l^ population, with a low percentage (2.63% Figure 5E 2.45% Figure 5H) of the CD209^h^ population observed. Similar percentages were observed in the presence of the NOX2 inhibitor. On the contrary, in the presence of both concentrations of CEL, the CD209^h^ population was enriched (43.9 and 69.8% Figure 5E, 18.1 and 41.3% Figure 5H). Together, these data suggest that NOX5, but not NOX2, is a downstream effector for Tofacitinib and partially reverses its inhibitory effect on Mo-DC differentiation.

## Discussion

In this study, we explored monocyte-derived dendritic cell differentiation and function in RA and PsA patients. We observed that monocytes were fully differentiated to Mo-DC in HC, PsA and RA when analyzed at the end of the differentiation process. However, we demonstrated that RA monocytes are already primed to become DC, as shown by the faster rate of differentiation and the presence of activation markers as early as 1 day post-differentiation. We demonstrated that RA Mo-DC were phenotypically different to that of PsA, displaying a more mature phenotype associated with altered cellular-morphology and dendrite formation. This was associated with significant suppression of monocyte-endocytosis and antigen-uptake mechanisms and a decreased ROS production. Tofacitinib inhibition of Mo-DC differentiation was paralleled by alteration in NOX5/NOX2 balance, an effect mediated through inhibition of NOX5, but not NOX2. Thus, we identify a novel mechanism of action whereby the inhibitory effect of Tofacitinib on Mo-DC differentiation is mediated through NOX5, a mechanism which might alter Mo-DC development.

CD209, a marker of differentiation, was significantly increased during differentiation in HC, RA and PsA, with no significant difference observed among the groups following 7 days differentiation. This was mirrored by a decrease in the monocytic marker CD14. The time-course experiment underlined, however, that the rate of differentiation was different among the groups, with the RA Mo-DC differentiating at a faster rate than that of HC and PsA Mo-DC, suggesting monocytes from RA patients are already in an activated or primed state. These results should be confirmed in a larger sample. This is consistent with a study by Smiljanovic et al., which highlighted distinct transcriptomic regulation between circulating RA monocytes (50), with more than 100 genes upregulated in RA, including chemokine receptors and genes involved in homeostasis. In addition, it has been shown that different inflammatory DC subsets were more active in RA, when compared to that of HCs (51, 52). Consistent with this, a newly discovered subset of inflammatory DC has been shown to present a distinct transcriptome which make them closer to BDCA1^+^ DCs and inflammatory macrophage than to that of monocytes (53); moreover, CD1c^+^ DCs and CD141^+^ DCs were demonstrated to have significantly increased cell surface expression of activation/maturation markers CCR7 and CD86 in early RA compared with HCs (51). The increased percentage of CD14^+^ monocyte cells observed in RA and, to a lesser extend in PsA, when compared to HC, suggest there are more circulatory monocytes in the blood of IA patients. Interestingly, the frequency of CD14^+^ monocytes was significantly decreased in the fluid of RA, and to a lesser extend of PsA, patients, suggesting CD14 cell infiltration in to the joint is minimal, or alternatively that these cells might have differentiated at the site of inflammation, into monocytic-derived cells, perhaps including DC. This is in line with our previous observation examining CD141^+^ DC cells, which were found enriched in the inflamed synovial joint of IA patients and transcriptionally distinct from their HC blood counterpart (10). The increase in chemokine receptors and IA CD14^+^ cells, when compared to HC, in particular CCR7 and CXCR4, suggested that monocytes might be primed and activated. In fact, although less CD14^+^ cells where observed in the fluid of IA patients, we observed a distinct chemokine receptor pattern between the blood and the fluid of IA patients, which could facilitate their infiltration and activation stage within the joint. Furthermore, a higher percentage of CXCR3 and CXCR5 was observed in RA synovial fluid monocytes when compared to the PsA, which might suggest a higher migratory capacity of CD14^+^ cells, indicative of a more mature and active phenotype (43, 44, 54). Further experiments are required to characterize Mo-DC in blood if IA patients and investigate their migratory capacity and their contribution to disease.”

The main function of dendritic cells in their immature stage, is the uptake and processing of antigens (45), which are subsequently presented to T cells; this function has been shown to be more efficient in Mo-DC, than in other conventional DC (55). Interestingly, antigen uptake was impaired in RA, compared to that of PsA and HC, where antigen uptake remained functional. This observation, along with the increased rate of differentiation observed for RA monocytes, suggests earlier activation of RA Mo-DC, which is further supported by the observed upregulation of CD40 and CD86, as early as 1 day post differentiation. The observation that GM-SCF receptor was demonstrated to be increased in both RA and PsA, suggests that the priming of RA monocytes is not due to an increase in the cytokine receptor; further analysis should investigate also the contribution of the IL4-R. This was phenotypically translated into alteration in the morphological appearance of RA monocytes, where an elongated morphology displaying visible dendrites was observed at this monocytic stage. Furthermore, similar to the lack of endocytosis observed in RA Mo-DC in this study, impaired phagocytic activity has previously been observed in neutrophils isolated from RA patients (56, 57). The early activation of Mo-DC from RA patients may alter their ability to uptake antigen and lead them to an early maturation and activation stage. Indeed, it has been observed that activated/mature Mo-DC decrease their ability to uptake antigen and switch their function to T-cell presentation (31, 58), suggesting RA Mo-DC might already express features of mature DC, at the end of Mo-DC differentiation. To investigate this hypothesis, further studies should compare RA and PsA Mo-DC T-cell activation at the end of the differentiation as well as at the end maturation stages. In addition, these experiments were all conducted on *in- vitro* generated Mo-DC, therefore future studies should confirm these data in blood DC. To the best of our knowledge, very few studies have compared DC from RA and PsA patients, both *in-vitro* and *ex-vivo*. This is certainly due to the technical difficulties to work with primary DCs isolated from patients or healthy individuals. Jongbloed et al. (11) compared myeloid and plasmacytoid DC from RA and PsA patients, showing they are both enriched into the inflamed joint where they show semi-mature features. In addition they show that both DC subsets respond to TLR stimulation, by producing TNFα and IL-10, although they were unable to stratify these results between the 2 diseases. It is well-established that RA and PsA synovial immunopathology differ in terms of blood vessel activation, T cell subsets, and cytokines profiles (9). In addition, while TNFi and abatacept are utilized for both arthropathies, they differ in their responses to anti-IL-17, anti-IL23, and anti-IL-6 (9). The role DC play in mediating these responses is not known. While studies exploring DC function in peripheral blood are challenging, examining their possible role in IA pathology within the synovium is even more complex. CD1c myeloid DC have previously been identified in RA synovial fluid and peripheral blood (59, 60). Interestingly, following co-culture of RA CD1c DC with autologous peripheral blood CD4+ T cells, T cells stimulated with synovial DC produced significantly higher levels of the Th17, Th1, and Th2 cytokines IL-17, IFNγ, and IL-4, respectively (60). As highlighted above, different T cells subsets have been associated with RA (Th1) and PsA (Th17), however this study suggests, at least *in-vitro* that RA synovial DC can drive Th17 and Th2 responses in addition to Th1 cells. This perhaps suggests that the contribution of synovial DC to T cell differentiation may also significantly rely on the synovial microenvironment to direct the differentiation of specific T cell subset. Of note, RA synovial DC also produced IL-23 and IL-12, cytokines required to drive Th17 and Th1 responses, respectively (60). All the aforementioned studies concentrate on myeloid DC, therefore further experiments are required to characterize Mo-DC in blood of IA patients and investigate how they contribute to disease.

An intricate network of cytokines is involved in the pathogenesis and progression of RA and PsA, which activate among others, the JAK-STAT pathway (22–24), which leads to the activation of multiple genes involved in driving inflammation in the inflamed joint. Moreover, the JAK/STAT pathway has been showed to be involved in DC development. Different STATs have been shown to be expressed during Mo-DC differentiation and maturation, with STAT1-3-5 and 6 phosphorylated during Mo-DC differentiation, and STAT1 and STAT3 being further activated during TLR maturation (61). Furthermore, the JAK1/2 inhibitor CYT387, has been shown repress DC differentiation and maturation, reducing their capacity to promote T cell differentiation (62), similarly the JAK2 inhibitor AG490 has also been shown to be involved in mediating DC maturation (63). In addition, our group has previously shown that the JAK/STAT pathway is one of the pathway involved in Mo-DC differentiation (8), and STAT3 inhibition has been shown to repress endothelial cells differentiation from DC (64). In this study, we show that JAK1/JAK3 selective inhibition by Tofacitinib strongly inhibited Mo-DC differentiation in all groups, suggesting its role in Mo-DC development. The inhibitor effect on Mo-DC differentiation was an early event in RA, as observed by the reduction of the CD209 marker at 1 day post differentiation, with no effect on PsA until 3 days post-differentiation. In addition, Tofacitinib also inhibited the expression of the activation marker, CD40 in RA monocytes, with a minimal effect observed on PsA monocytes. The response to Tofacitinib is not the same across all therapeutic targets in IA, with studies showing a lack of effect of TNF or IL-6 blockade on DC physiology in antigen-induced arthritis (AIA) in mice (20). Here we show that Tofacitinib inhibition of Mo-DC differentiation translate into a functional impairment, as observed by the inhibition of both receptor-mediated endocytosis and macropinocytosis in HC and PsA, and to a lesser extent, in RA This effect may be mediated, in part, through the observed decrease in CD209 expression in response to Tofacitinib, consistent with previous studies which have demonstrated that the presence of CD209 on the surface of DC is required for DC antigen uptake (65).

Several studies, including ours, have highlighted the importance of NADPH oxidases (NOX) expression in Mo-DC differentiation and antigen presentation (8, 37, 66). In particular, our previous study identified a significant induction of NOX5, mirrored by a decrease in NOX2 expression during Mo-DC differentiation (8). In addition, it showed the involvement of NOX5 in Mo-DC differentiation by altering, among others, the JAK/STAT pathway (8). Therefore, we postulated that JAK/STAT inhibition of differentiation observed in the current study, was a result of an altered balance of NOX5/NOX2 expression. Indeed, we observed a switch in the expression of NOX5 and NOX2 expression, with high level of NOX2 and reduced level of NOX5 in the presence of Tofacitinib. Previous studies have shown a key role for ROS produced by NADPH in the alkalinisation of the phagosome in Mo-DC (37, 55, 66, 67), necessary for a tight controlled antigen uptake. Therefore, the reduction of NOX5-dependent ROS release by Tofacitinib in PsA suggested a direct functional effect on NOX5, which can be linked to the reduced endocytosis observed in Mo-DC in the presence of Tofacitinib. Therefore, JAK1/3 inhibition on ROS and NADPH oxidase expression is translated into a functional impairment in Mo-DC. In addition, impaired ROS production, observed in RA Mo-DC, when compared to PsA, suggests that the altered endocytosis in RA Mo-DC is possibly due to their lack of ROS production. This is highlighted also by the lack of response of Mo-DC from RA patients to respond efficiently to ionomycin, suggesting an altered ROS production in these cells. Further study could investigate the mechanisms involved in these divergent responses. Although RA Mo-DC were not fully efficient in endocytosis, we did observe inhibition by Tofacitinib, where a trend in decreasing endocytosis below basal levels in RA was demonstrated.

The direct involvement of NOX5 as a downstream effector of JAK1/3 inhibition, was confirmed by pharmacological inhibition of NOX5 and NOX2, where we observed a loss of effect of Tofacitinib in the presence of the NOX5 inhibitor, but not NOX2 inhibitor, suggesting NOX5 is a substrate for Tofacitinib and it is required for its mechanism of action. Although it has been shown that Celastrol not only inhibits NOX5, but can also inhibit NOX2 (8, 49), the fact that we show a lack of effect of the specific NOX2 inhibitor (TAT) on Tofacitinib mechanisms, suggests that the effect observed in this study is solely due to NOX5 inhibition and not NOX2. We have previously shown in Mo-DC that NOX5 inhibition, both pharmacologically and with siRNA technology, significantly decrease STAT5 phosphorylation (8), in addition it has been shown that STAT5 phosphorylation is inhibited by Tofacitinib in T cells (68) and lymphocytes from RA patients (69). We can, therefore, speculate, that STAT5 might be involved in the Tofacitinib/NOX5 pathway in Mo-DC from RA and PsA patients. While in this study we have examined the effect of Tofacitinib on Mo-DC differentiation, selective inhibition of JAK1 or JAK3, would further clarify which of the two JAKs is responsible and which specific downstream STAT(s), is responsible for the specific effect of Tofacitinib on Mo-DC development that is observed in this study.

**Figure 6:**
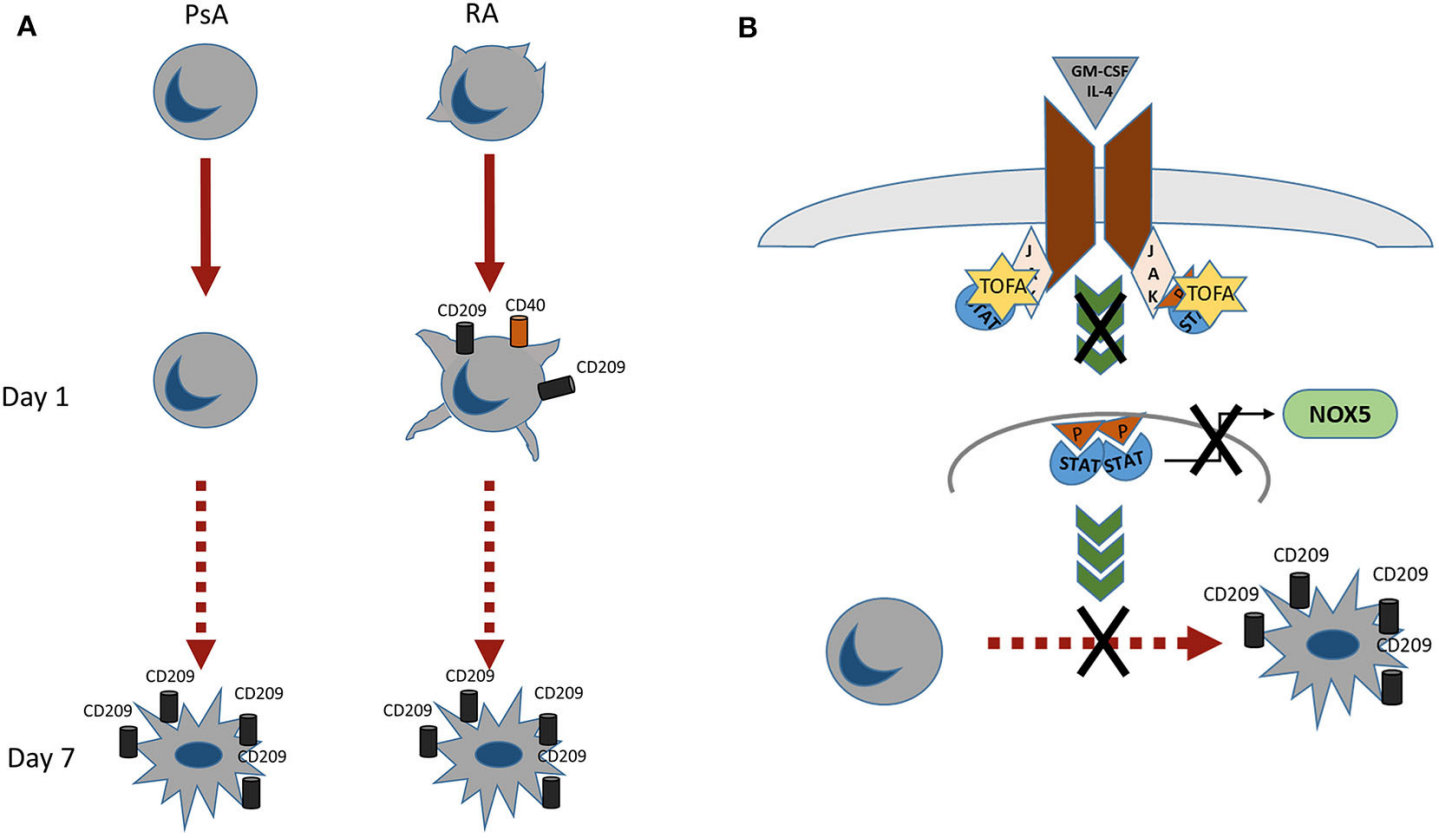
**(A)** RA monocytes exhibits morphological changes which are enhanced already 1 day post-differentiation, where an increase in CD209 differentiation marker and CD40 activation marker are observed. At day 7 post-differentiation both RA and PsA express similar level of the differentiation marker CD209. **(B)** Proposed mechanism of action of Tofacitinib on Mo-DC differentiation. Tofacitinib, by inhibiting the JAK pathway, leads to a decrease of NOX5 and a subsequent decrease of Mo-DC differentiation.

In conclusion we observed different morphological and functional profiles between monocytes obtained from RA and PsA patients (Figure 6A), with RA monocytes being primed to differentiate into DC and exhibiting differentiation and activation markers at earlier time point compared to that of PsA. In addition, we demonstrated a novel mechanism of Tofacitinib in RA and PsA patients (Figure 6B), which is partly mediated by NOX5 and ROS production. All together, these data suggest a differential behavior of monocytes from RA and PsA patients when differentiating into Mo-DC and an impairment development of these cells in the presence of Tofacitinib.

## Data Availability Statement

All datasets generated for this study are included in the article/Supplementary Material.

## Ethics Statement

The studies involving human participants were reviewed and approved by St. Vincent's University Hospital ethics committee RS18-055 and Tallaght University Hospital ethics committee 2017-06-21. The patients/participants provided their written informed consent to participate in this study.

## Author Contributions

VM and UF designed the experiments. VM, MC, and AF performed experiments. SW helped in patient recruitment and processing. DV and CL recruited the patients. VM analyzed the data. VM and UF wrote the manuscript with all authors contributing to writing. UF supervised the project. All authors contributed to the article and approved the submitted version.

## Conflict of Interest

The authors declare that the research was conducted in the absence of any commercial or financial relationships that could be construed as a potential conflict of interest.

## Supplementary Material

Supplementary Table 1 Patients demographic. Age, Gender, CRP, VAS and serology. Details on treatments are shown. None of the patients were on Tofacitinib treatment at the time of the sample acquisition. Healthy control (HC) demographics (Age, Gender).

Supplementary Figure 1 Gating strategy for the identification of CD14^+^ cells in PBMC and SFMC from PsA and RA patients. Cells were gated based on forward and side scatter and dead cells and doublets were removed. Live Dead Near-IR (Molecular Probes) was used to eliminate dead cells. The cells were then gated as CD45^+^/LIN^−^(CD19-CD20-CD56-CD3)/HLA-DR^+^/CD14^+^. Chemokines receptors expression was identified by comparison with an FMO (Fluorescence Minus One Control).

Supplementary Figure 2 GM-CSF receptor expression in the CD14^+^ population. **(A)** Dot plot for GM-CSF R percentage expression in the CD14^+^ population and representative flow plot for HC, PsA, and RA patients. FMO (Fluorescence Minus One Control). **(B)** MFI for GM-CSF R and representative histograms. Data are shown as Mean ±SEM (HC *n* = 3, PsA *n* = 7. RA *n* = 7). One-Way ANOVA ^*^*p* < 0.05, (vs. HC).

Supplementary Figure 3 **(A)** SPICE algorithm flow cytometric analysis of peripheral blood of HC (*n* = 5), PsA (*n* = 9) and RA (*n* = 14) and synovial fluid from PsA (*n* = 5) and RA patient (*n* = 9) CD14+ cell expression of the chemokines receptors CCR6, CCR7, CXCR3, CXCR4 and CXCR5. **(B)** Data are represented as mean ±SEM and differences among groups were evaluated with two-way ANOVA ^*^*p* < 0.05, ^**^*p* < 0.01, ^**^*p* < 0.001 (PBMC vs SFMC).

Supplementary Figure 4 **(A)** Monocytes from HC (*n* = 7) were differentiated with GM-CSF/IL-4 in the presence or absence of Tofacitinib 1μM (or DMSO) 7 day post-differentiation. CD209, surface differentiation marker, was evaluated by flow cytometry in the CD11c+ population. Mean ±SEM and representative histograms. **(B)** ROS production in HC (*n* = 3, Kruskal-Wallis test ^***^*p* < 0.001 IONO vs IONO+TOFA as per Figure 5). **(C,D)** Pharmacological inhibition of NOX_5_ and NOX_2_. Inhibition of NOX_5_ by CEL (1 and 2 μM) and NOX2 by TAT (15 μM) 5 minutes before Tofacitinib treatment (C-1μM top and D-0.5 μM) and cytokines stimuli. Average of HC *n* = 3 separate experiments is represented. ^*^*p* < 0.05, ^**^p<0.01, ^***^*p* < 0.001.
