# Supplementary material for: Monocyte-Derived Dendritic Cell Differentiation in Inflammatory Arthritis Is Regulated by the JAK/STAT Axis via NADPH Oxidase Regulation
Source: Front Immunol. 2020 Jul 7;11:1406. doi: 10.3389/fimmu.2020.01406 (PMC7358435; doi:10.3389/fimmu.2020.01406)

Supplementary Table 1

Parameters	HC (n=13)	RA (n=35)	PsA (n=26)
Age (Mean +/- SD)	38 (+/-12)	57 (+/-17)	52 (+/-12)
**Gender**			
Male	38%	36%	35%
Female	62%	64%	65%
**CRP**		15(+/-21)	7.7 (+/-7.3)
**VAS**		45 (+/-30)	42(+/-21)
**ACPA** POS		77%	0%
NEG		23%	100%
**RF** POS		63%	0%
NEG		37%	100%
**Treatments**			
No Medication		21%	28%
Methotrexate alone		24%	40%
MTX in combination		27%	0%
Anti-TNF		6%	20%
Steroid		15.0%	0%
Others		7.0%	12.0%

Supplementary Figure 1

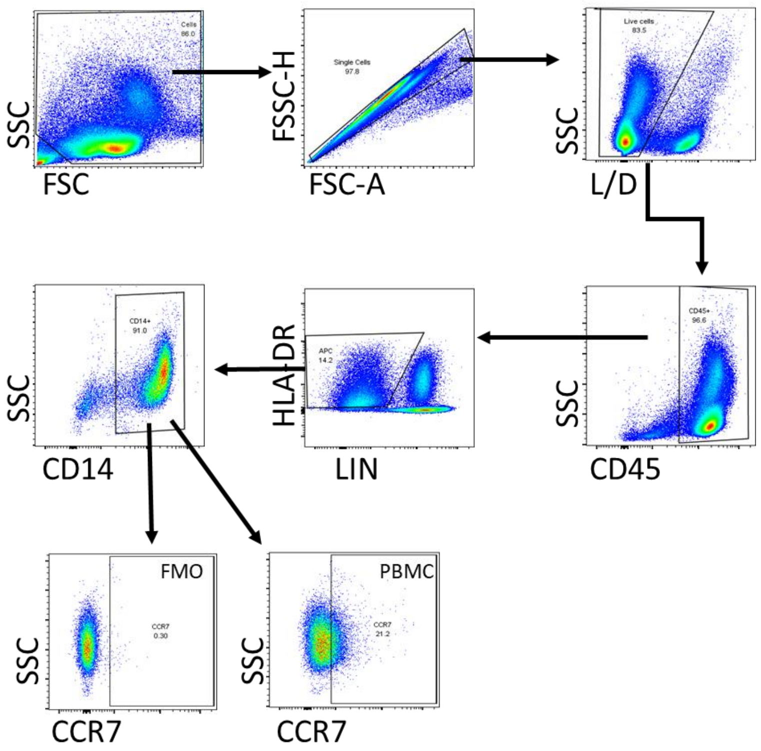

Supplementary Figure 2

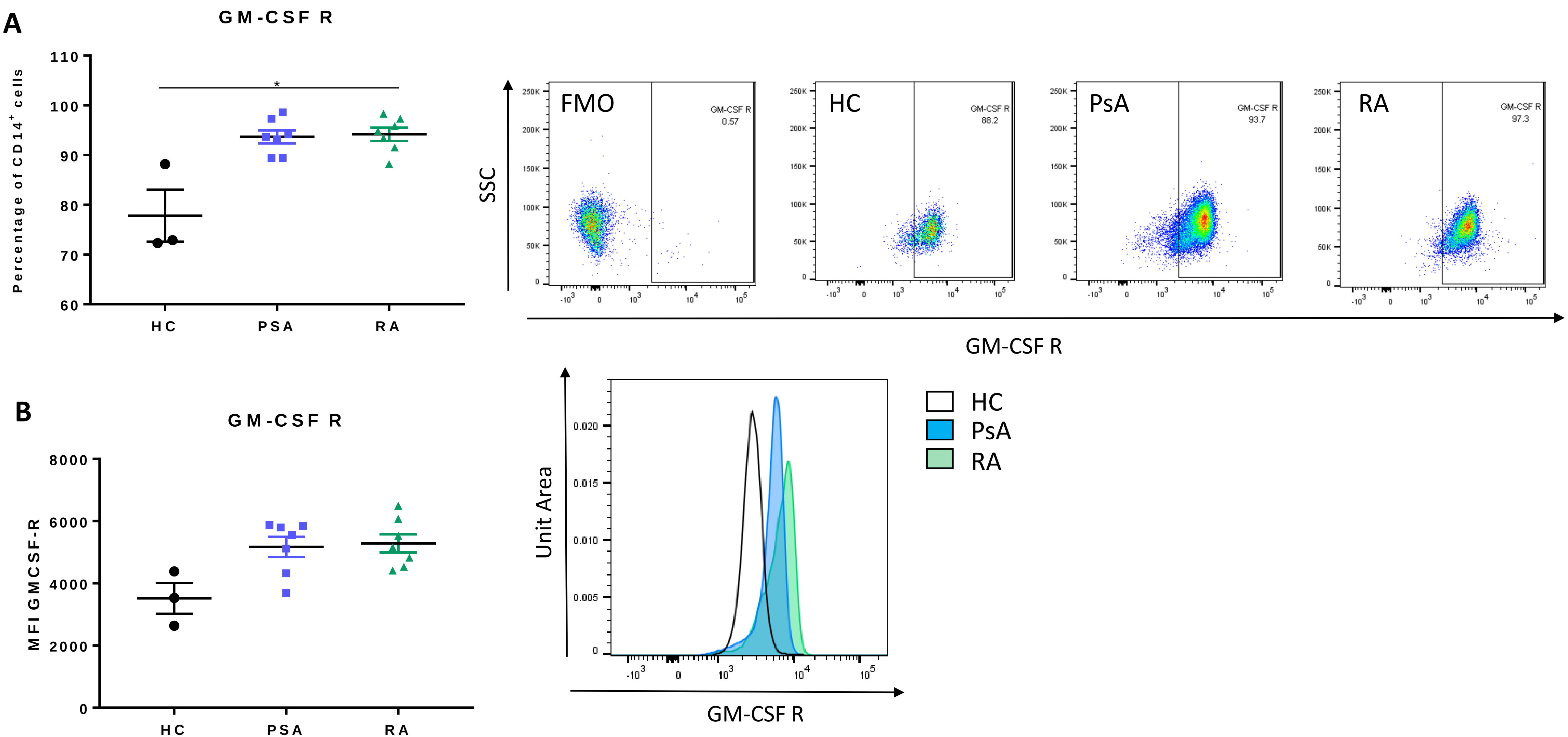

Supplementary Figure 3

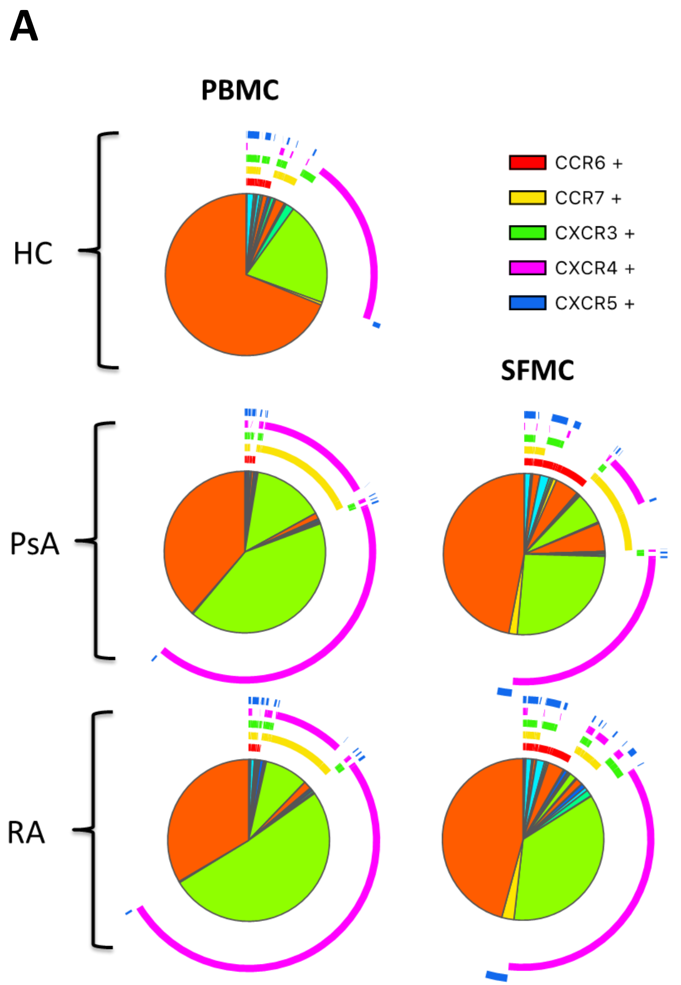

#	CCR6	CCR7	CXCR3	CXCR4	CXCR5
1	+	+	+	+	+
2	+	+	+	+	-
3	+	+	+	-	+
4	+	+	+	-	-
5	+	+	-	+	+
6	+	+	-	+	-
7	+	+	-	-	+
8	+	+	-	-	-
9	+	-	+	+	+
10	+	-	+	+	-
11	+	-	+	-	+
12	+	-	+	-	-
13	+	-	-	+	+
14	+	-	-	+	-
15	+	-	-	-	+
16	+	-	-	-	-
17	-	+	+	+	+
18	-	+	+	+	-
19	-	+	+	-	+
20	-	+	+	-	-
21	-	+	-	+	+
22	-	+	-	+	-
23	-	+	-	-	+
24	-	+	-	-	-
25	-	-	+	+	+
26	-	-	+	+	-
27	-	-	+	-	+
28	-	-	+	-	-
29	-	-	-	+	+
30	-	-	-	+	-
31	-	-	-	-	+
32	-	-	-	-	-

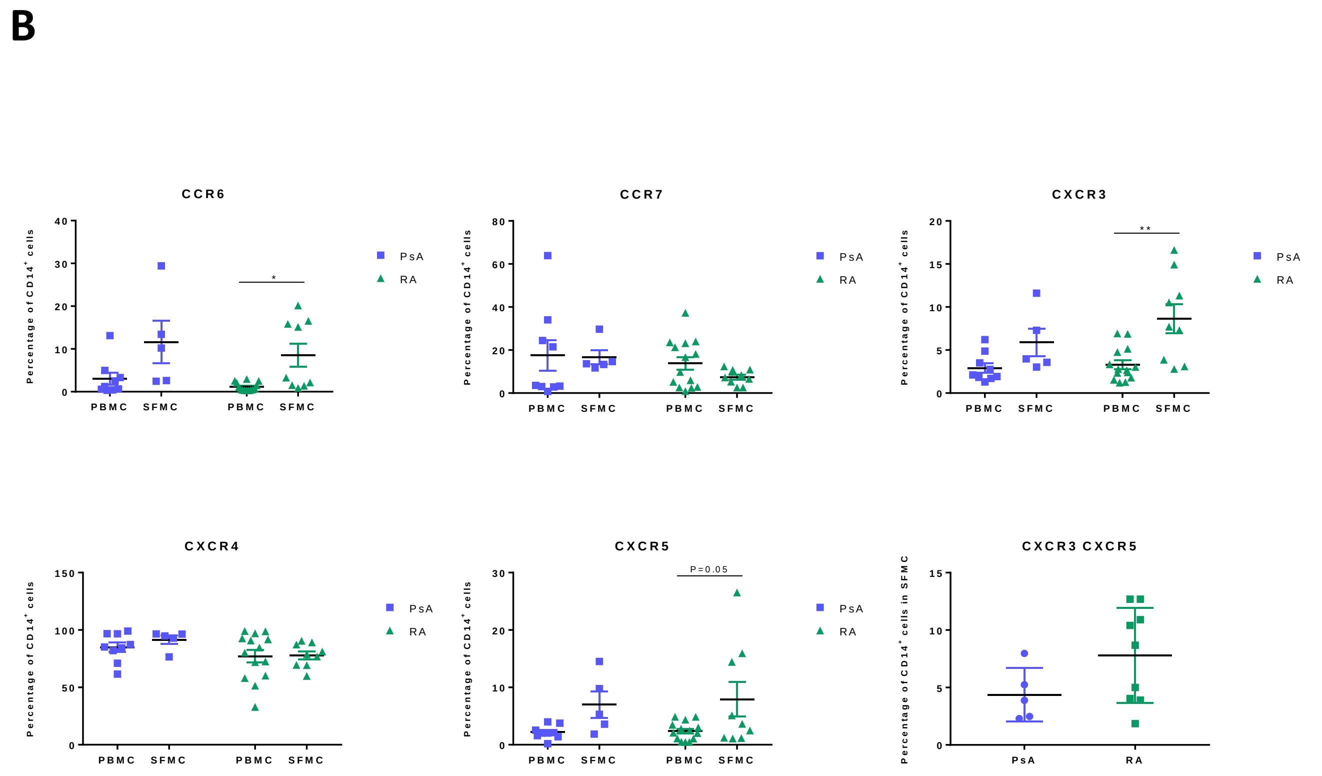

Supplementary Figure 4

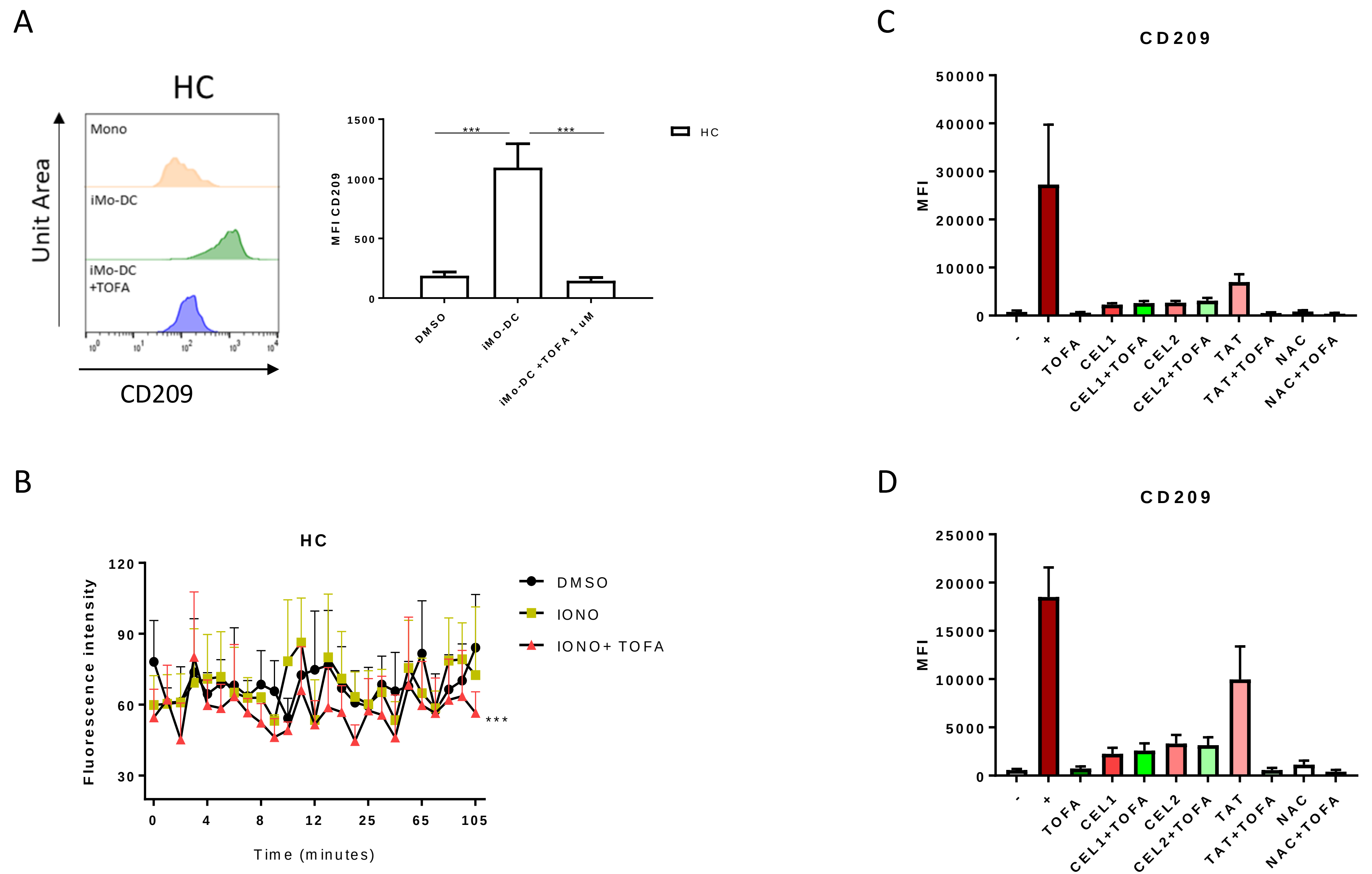

Supplement: Supplementary Table 1 — Patients demographic. Age, Gender, CRP, VAS and serology. Details on treatments are shown. None of the patients were on Tofacitinib treatment at the time of the sample acquisition. Healthy control (HC) demographics (Age, Gender). [file Presentation_1.PDF]
